# The Prevalence and Risk Factors of Acute Kidney Injury during Colistin Therapy: A Retrospective Cohort Study from Lebanon

**DOI:** 10.3390/antibiotics12071183

**Published:** 2023-07-13

**Authors:** Rima Moghnieh, Rola Husni, Mariana Helou, Dania Abdallah, Loubna Sinno, Marwa Jadayel, Kawsar Diab, Carmen Chami, Marah Al Rachid, Diana Caroline Awad, Aline Zaiter, Mohamed H. Sayegh

**Affiliations:** 1 Department of Internal Medicine, Lebanese American University Medical Center, 13-5053 Beirut, Lebanon; roula.samaha@laumcrh.com (R.H.); mariana.helou@laumcrh.com (M.H.); 2 Department of Internal Medicine, Division of Infectious Diseases, Makassed General Hospital, 11-6301 Beirut, Lebanon; 3 Pharmacy Department, Makassed General Hospital, 11-6301 Beirut, Lebanon; rpdania@gmail.com; 4 Department of Medical Research, Makassed General Hospital, 11-6301 Beirut, Lebanon; loubna_lms@hotmail.com; 5 Faculty of Pharmacy, Lebanese University, 6573/14 Beirut, Lebanon; marwajadayel.1@gmail.com; 6 Department of Obstetrics and Gynecology, Al-Zahraa Hospital-University Medical Center, VF7P+JVR Beirut, Lebanon; kawsar.diab@zhumc.org.lb; 7 Faculty of Medicine, Lebanese University, 6573/14 Beirut, Lebanon; carmenmchami@hotmail.com (C.C.); marahalrachid95@gmail.com (M.A.R.); diana94caroline@hotmail.com (D.C.A.); aline.zaiterr@gmail.com (A.Z.); 8 Faculty of Medicine, American University of Beirut, 11-0236 Beirut, Lebanon; msayegh@aub.edu.lb

**Keywords:** acute kidney injury, colistimethate sodium, KDIGO, Lebanon

## Abstract

Introduction: The current study aimed to determine the prevalence, risk factors, and stages of severity of acute kidney injury (AKI) caused by colistimethate sodium (CMS) treatment in patients diagnosed with systemic antibiotic-resistant Gram-negative bacterial infections. The predictors of all-cause mortality in this patient population were also examined. Methods: This retrospective cohort study included patients who were admitted to a university-affiliated hospital and acute tertiary care referral center in Beirut, Lebanon between January 2015 and December 2018 and underwent CMS treatment for a period of 48 h or more. Results: The study sample included 298 adult patients, of which 46.3% (*n* = 138/298) developed AKI (assessed using the Kidney Disease Improving Global Outcomes (KDIGO) criteria). Of these, 37.7% (*n* = 51/138) were diagnosed with stage 1 AKI, 23.9% with stage 2 (*n* = 33/138), and 38.4% with stage 3 (*n* = 53/138). Nephrotoxicity was reversed in 87.5% of AKI patients who survived until hospital discharge. Independent risk factors for AKI included patient age ≥ 75 years (aOR = 1.854; 95% CI: 1.060–3.241; *p*-value = 0.03); underlying chronic kidney disease (aOR = 4.849; 95% CI: 2.618–9.264; *p*-value < 0.0001); and concomitant use of vasopressors (aOR = 4.305; 95% CI: 2.517–7.456; *p*-value < 0.0001). Multivariate analysis showed that the predictors of severe AKI (stage 2 or 3) included baseline hypoalbuminemia (aOR = 2.542; 95% CI: 1.000–6.564; *p*-value = 0.05); concomitant use of vasopressors (aOR = 6.396; 95% CI: 2.741–15.87; *p*-value < 0.0001); and CMS days of therapy (DOT) prior to development of AKI ≥ 7 days (aOR = 4.728; 95% CI: 2.069–11.60; *p*-value < 0.0001). All-cause mortality was recorded in 51.3% of patients (*n* = 153/298), and this was significantly higher in patients with AKI (76.8%; *n* = 106/138) compared to those without (29.4%; *n* = 47/160; OR = 7.964; 95% CI: 4.727–13.417; *p*-value < 0.0001). Independent predictors of all-cause mortality included a baseline Charlson comorbidity index score ≥5 (aOR = 4.514; 95% CI: 2.443–8.530; *p*-value < 0.0001); concomitant use of vasopressors (aOR = 7.76; 95% CI: 4.238–14.56; *p*-value < 0.0001); and CMS-induced AKI (aOR = 4.117; 95% CI: 2.231–7.695; *p*-value < 0.0001). Conclusions: The findings of this study suggest that old age, history of chronic kidney disease, and concomitant vasopressor treatment are all independent predictors of CMS-induced AKI. The risk of developing severe AKI significantly increases with CMS DOT. Understanding the risk factors of nephrotoxicity is essential for improving prognosis and treatment outcomes.

## 1. Introduction

The prevalence of antibiotic resistance has risen globally, although the true scale of this health crisis has been hard to understand or quantify [1]. The turn of the 21st century was characterized by the rapid emergence of antibiotic resistance among many Gram-negative bacilli such as *Acinetobacter baumannii*, *Pseudomonas* spp., *Klebsiella* spp., and other Enterobacterales [2,3]. Extensive efforts to identify new drugs that counteract the mechanisms underlying this phenomenon have been largely unsuccessful, and the existing antimicrobial armamentarium is still inadequate in comparison to the natural survival instinct of these microorganisms.

The increasing burden of antibiotic resistance, evidenced by high morbidity and mortality rates, has necessitated efforts to identify new antibiotics or re-introduce old ones (e.g., polymyxins such as colistin) into clinical practice [2,3]. Polymyxins were first discovered in the late 1940s, and their use was later discontinued due to the increasing incidence of associated side effects such as nephrotoxicity and neurotoxicity. However, despite these limitations, they were re-introduced into clinical practice in the early 2000s due to their potent bactericidal effects [2,3].

The spread of highly resistant bacteria that lead to severe systemic infections and the limited availability of antibiotics that can address them adequately have made colistin an increasingly popular option over the past two decades [4,5]. Lebanon, a Middle Eastern country with limited resources, has been particularly affected by the spread of antibiotic resistance since the start of the 21st century [6].

The rates of antibiotic resistance in Lebanon are submitted annually to the World Health Organization (WHO) Global Antimicrobial Resistance and Use Surveillance System (GLASS). In 2020, the imipenem resistance rate reached 92% among *Acinetobacter species* causing bacteriologically confirmed bloodstream infections, and among Enterobacterales, it ranged between 51 and 67% [7]. In comparison with similar data from the WHO Eastern Mediterranean Region as a whole, the median imipenem resistance among *Acinetobacter species* causing bacteremia was lower than that reported from Lebanon during 2020, reaching 75.3% (interquartile range (IQR), 60.5–85.6%)) [8]. Similarly, among Enterobacterales, imipenem resistance rates were lower than those reported from Lebanon with a median of 6.2% in *Escherichia coli* (IQR, 2.1–15.1%) and 36.1% in *Klebsiella pneumoniae* (IQR, 17.5–53.6%) [8]. It is worth noting that, on the global scale, the median imipenem resistance rates were 69% (IQR, 30.8–85.0%) in *Acinetobacter species* causing bacteremia, 0.5% in *Escherichia coli* (IQR, 0.0–5.3%), and 8.4% in *Klebsiella pneumoniae* (IQR, 0.6–36.6%), according to the 2020 WHO GLASS data [8].

Although various new treatment combinations (e.g., β-lactam antibiotics + potent β-lactamase inhibitors (BLI) with anti-carbapenemase activity) have been introduced since, the associated high costs have made them difficult to access, particularly in low resource settings [9]. Consequently, affordable and potent drugs such as colistin have become increasingly popular, particularly among patients diagnosed with difficult-to-treat infections caused by resistant bacteria [6,10].

The current study aimed to determine the prevalence, risk factors, and stages of severity of acute kidney injury (AKI) caused by colistimethate sodium (CMS) treatment in patients diagnosed with systemic antibiotic-resistant Gram-negative bacterial infections and admitted to a tertiary care hospital in Lebanon. Additionally, the predictors of all-cause mortality in this patient population were also examined.

## 2. Results

### 2.1. Patient Characteristics

The study sample included 298 adult patients, of which 44% were male. The study sample had a median age of 69 years (interquartile range (IQR): 51–80 years; Table 1), and 57.7% of the patients exhibited a baseline Charlson comorbidity index (CCI) score ≥ 5 (median CCI score: 5; IQR: 2–7). The most frequently observed comorbidities were cardiovascular disease (54.3%); chronic kidney disease (50.7%); malignancy (31.9%); and type 2 diabetes mellitus (31.5%). Median baseline serum albumin levels were 3.01 g/dL (IQR: 2.5–3.7 g/dL), and hypoalbuminemia (<3.5 g/dL) was observed in 66.4% of patients (median baseline serum albumin level: 3.01 g/dL, IQR: 2.5–3.7 g/dL).

The majority (57.7%) of patients were admitted to the regular ward when CMS treatment was initiated, while the remaining 42% were admitted to the critical care unit. Hypotension and vasopressor use was observed in 46.6% of patients, while 57% of patients were on mechanical ventilation during CMS therapy (Table 2).

CMS was prescribed mainly for the treatment of nosocomial pneumonia (39.6%); sepsis (33.2%); bacteremia (11.1%); and febrile neutropenia (11.1%; Table 1). Causative organisms were reported in 61.4% of the cases, and the majority of them were drug-resistant and/or carbapenem-resistant Gram-negative bacteria (55%). These included carbapenem-resistant and extensive drug-resistant *Acinetobacter baumannii* isolated in 44.3% of patients, and carbapenem-resistant *Pseudomonas aeruginosa* isolated in 9.4% of patients. Colistin was mostly prescribed in combination with other antibiotics (86%; Table 2), and a loading dose was given to 77.5% of patients (of which 66.8% received a loading dose of 9 million international units (MIU)).

Concomitant use of other nephrotoxic drugs was observed in 81.2% of patients, of which 48.7% received two or more drugs in addition to CMS (Table 2). These included non-antimicrobial agents such as vasopressors (49%), diuretics (37.6%), and radio-contrast agents (11.7%), as well as antimicrobials such as vancomycin (12.1%) and valacyclovir (11.4%; Table 2).

### 2.2. Prevalence of CMS-Induced AKI, Disease Severity, Renal Recovery, and Degree of Reversibility

**Figure 1:**
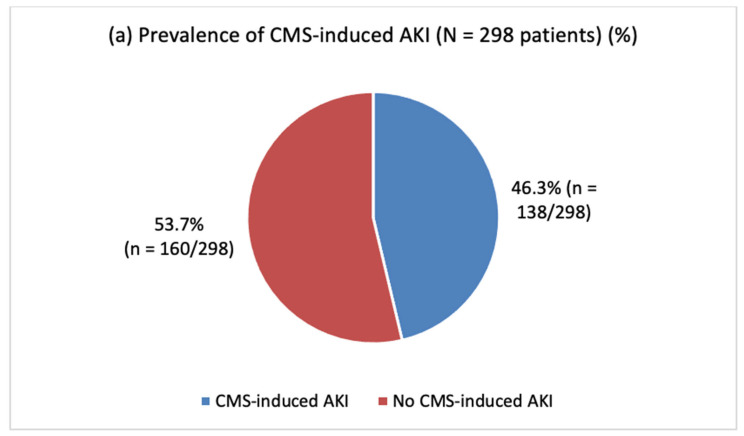

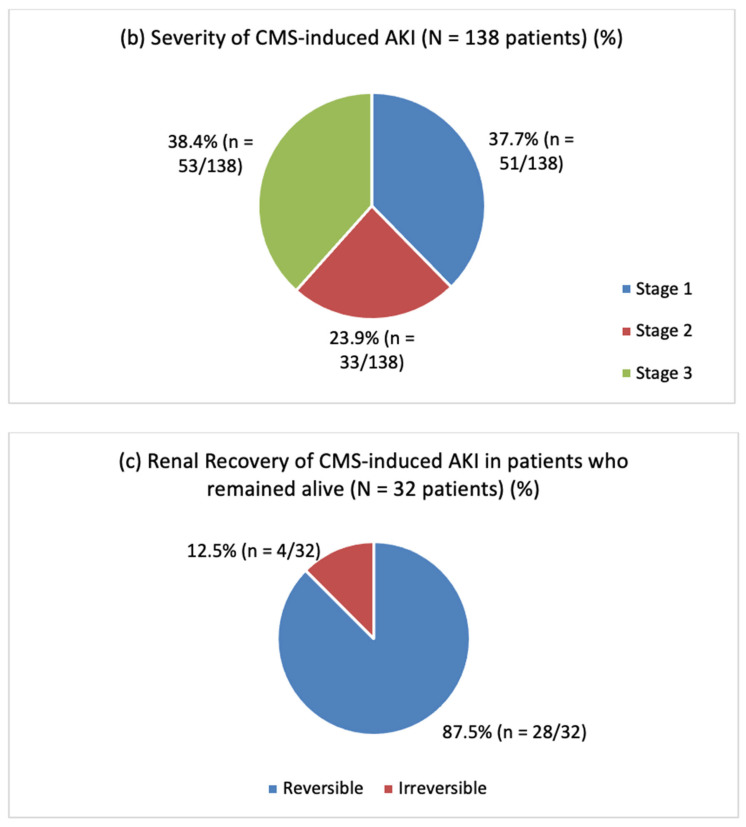

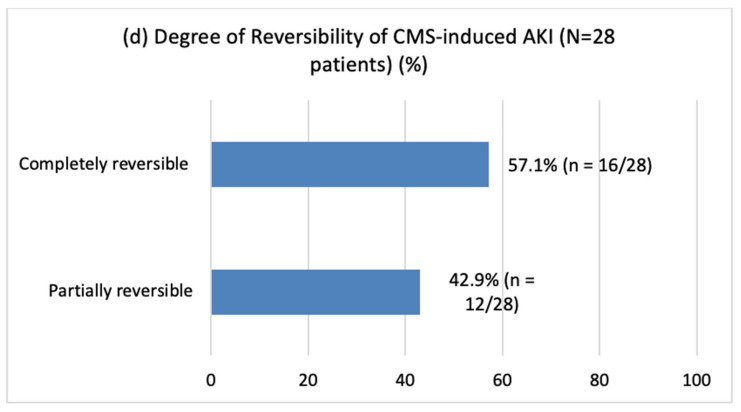
Prevalence (**a**), severity (**b**), renal recovery (**c**), and degree of reversibility (**d**) of CMS-induced AKI. Abbreviations: AKI = acute kidney injury, CMS = colistimethate sodium.

AKI was observed in 46.3% (*n* = 138/298) of patients receiving CMS treatment (Figure 1a), of which 37.7% (*n* = 51/138) were stage 1; 23.9% (*n* = 33/138) were stage 2; and 38.4% (*n* = 53/138) were stage 3 (Figure 1b). Reversal of nephrotoxicity was observed in 87.5% (*n* = 28/32) of patients with AKI who survived until hospital discharge, of which 57.1% were completely reversible at stage 1 AKI (*n* = 16/28) and 42.9% were partially reversible at stage 2 or 3 AKI (*n* = 12/28; Figure 1c,d).

### 2.3. Predictors of CMS-Induced AKI

Initial bivariate analysis was carried out to compare demographic characteristics; the presence of comorbidities and underlying conditions; indications for and causative organisms necessitating CMS use; and concomitant utilization of other nephrotoxic drugs between patients with and without AKI during hospitalization (Supplementary Table S1). Variables identified as being statistically significant were included in further multivariate analysis, and the findings showed that patient age ≥ 75 years (adjusted odds ratios (aOR) = 1.854; 95% Confidence Interval (CI): 1.060–3.241; *p*-value = 0.030); the presence of chronic kidney disease (aOR = 4.849; 95% CI: 2.618–9.264; *p*-value < 0.0001); and the concomitant use of vasopressors during CMS treatment (aOR = 4.305; 95% CI: 2.517–7.456; *p*-value < 0.0001) were independent risk factors for the development of AKI (Table 3).

### 2.4. Predictors of Severe CMS-Induced AKI (Stage 2 or 3)

Initial bivariate analysis was carried out to identify factors contributing to the development of severe CMS-induced AKI, and variables identified as being statistically significant were included in further multivariate analysis (Supplementary Table S2). The findings showed that baseline hypoalbuminemia (<3.5 g/dL; aOR = 2.542; 95% CI: 1.000–6.564; *p*-value = 0.049); concomitant use of vasopressors during CMS therapy (aOR = 6.396; 95% CI: 2.741–15.87; *p*-value < 0.0001); and CMS days of therapy (DOT) prior to AKI ≥ 7 (aOR = 4.728; 95% CI: 2.069–11.60; *p*-value < 0.0001) were independent risk factors predisposing for severe CMS-induced AKI (Table 4).

### 2.5. Association between AKI Severity and Renal Recovery by CMS DOT

Subgroup analysis showed that 43.5% (*n* = 60/138) of patients developed AKI > 7 days after initiation of CMS treatment (Table 3). The risk of severe AKI significantly increased with DOT, with 39.1% (*n* = 18/46) of patients developing it within 2 days of treatment initiation; 59.4% (*n* = 19/32) developing it between 3 and <7 days of treatment; and 81.7% (*n* = 49/60) developing it after ≥7 days of treatment (*p*-value < 0.0001; Table 5).

### 2.6. Prevalence and Predictors of All-Cause Mortality during CMS Treatment

All-cause mortality was observed in 51.3% (*n* = 153/298) of patients, and this proportion was significantly higher among those who developed AKI (76.8%; *n* = 106/138) compared to those who did not (29.4%; *n* = 47/160; OR = 7.964; 95% CI: 4.727–13.417; *p*-value < 0.0001; Supplementary Table S3). Furthermore, the prevalence of all-cause mortality was significantly higher in patients with severe AKI (83.7%; *n* = 72/86) compared to those with stage 1 AKI (65.4%; *n* = 34/52; OR = 2.723; 95% CI: 1.213–6.113; *p*-value = 0.01; Supplementary Table S3).

Initial bivariate analysis was carried out to compare demographic characteristics; the presence of comorbidities and underlying conditions; indications for and causative organisms necessitating CMS use; cumulative CMS dose in MIU and DOT; concomitant utilization of other nephrotoxic drugs; presence/absence of CMS-induced AKI; and disease severity between patients who died in the hospital and those who survived (Supplementary Table S3). Variables identified as being statistically significant were included in further multivariate analysis, and the findings showed that a baseline CCI score ≥ 5 (aOR = 4.514; 95% CI: 2.443–8.530; *p*-value < 0.0001); the concomitant use of vasopressors during CMS treatment (aOR = 7.76; 95% CI: 4.238–14.56; *p*-value < 0.0001); and the presence of CMS-induced AKI (aOR = 4.117; 95% CI: 2.231–7.695; *p*-value < 0.0001) were independent predictors of all-cause mortality in this patient population (Table 6).

### 2.7. Survival Analysis in Patients Undergoing CMS Treatment

**Figure 2:**
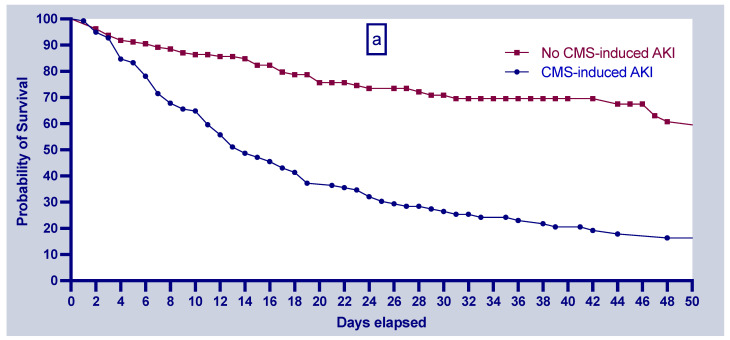

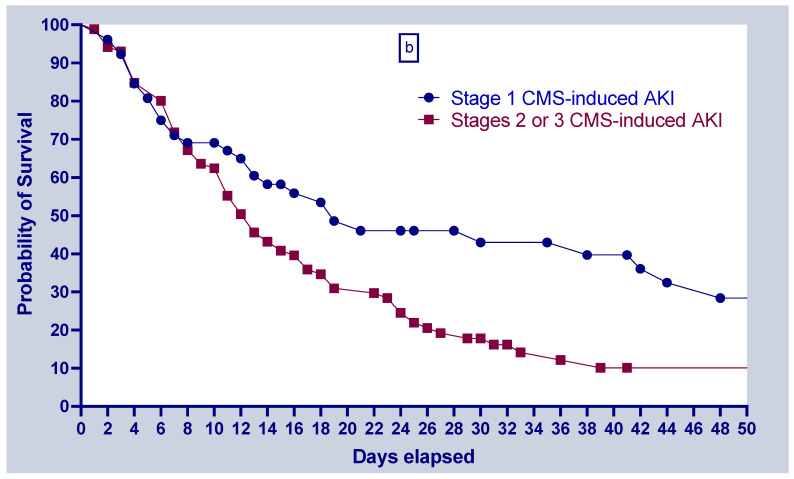
Kaplan–Meier survival analysis between (**a**) the CMS-induced AKI group (blue line with dots) and the no-CMS-induced AKI group (red line with squares) (log-rank test *p* < 0.0001); and (**b**) stage 1 CMS-induced AKI group (blue line with dots) and stage 2 or 3 CMS-induced AKI group (red line with squares) (log-rank *p* = 0.03). Abbreviations: AKI = acute kidney injury, CMS = colistimethate sodium.

Figure 2a shows the Kaplan–Meier survival curve for patients diagnosed with CMS-induced AKI. The probability of survival on day 7 of CMS treatment was 71.5% and 89.4% for patients with and without CMS-induced AKI, respectively. The corresponding proportions on day 14 were 49.2% and 83.9%, while those on day 28 were 28% and 71.9%. Further comparison of the two groups showed that patients without nephrotoxicity exhibited higher cumulative survival rates (log-rank test *p*-value < 0.0001; Figure 2a).

Figure 2b shows the Kaplan–Meier survival curve by disease severity. The findings showed that the probability of survival on day 7 of CMS treatment was 70% for patients with AKI stage 2 or 3 and 71% for patients with AKI stage 1. The corresponding proportions on day 14 were 45.7% and 59.6%, while those on day 28 were 18.4% and 46.7%. Further comparison by the severity of CMS-induced AKI showed that patients diagnosed with stage 2 or 3 exhibited lower cumulative survival rates than those diagnosed with stage 1 (log-rank test *p*-value = 0.03; Figure 2b).

## 3. Discussion

### 3.1. AKI Prevalence and Risk Factors

The findings of the current study showed that 46.3% of patients receiving CMS treatment developed AKI. Although this proportion may be a slight overestimation due to the advanced age of the study cohort and the presence of numerous comorbidities such as chronic kidney disease, it falls within the ranges reported previously [11,12,13]. Moreover, considering that CMS is typically used as a last-resort approach for severe infections, it is likely that the current study sample is representative of the general population requiring this mode of treatment. The International Consensus Guidelines for the Optimal Use of Polymyxins, published in 2019, estimated that the incidence rate of kidney injuries caused by polymyxin treatment ranged between 20 and 50% [14]. Previous studies reported similar prevalence rates ranging between 0 and 60% [12], while a recent systematic review by Wagenlehner et al., found that the prevalence of polymyxin-induced nephrotoxicity (assessed using the Kidney Disease Improving Global Outcomes (KDIGO) criteria) was approximately 34.7% (*n* = 783/2390 patients from 20 studies; 95% CI: 27.0–43.3%) [12]. These differences in reported prevalence rates can likely be attributed to variations in the study populations (e.g., demographic characteristics, comorbidities, and disease severity); the diverse internationally recognized criteria for defining kidney injury; the sensitivity of the diagnostic criteria used; colistin dosage and duration of treatment; and the presence of potential confounders such as the concomitant use of other nephrotoxic drugs [11,12,13].

In the current study, patient age ≥ 75 years, a history of chronic kidney disease, and the concomitant use of vasopressors during CMS treatment were independent risk factors for AKI, and these findings were in agreement with previous studies [11,12,13]. Elderly patients undergo several physiological changes that can limit nephron and kidney function, thus making this population particularly vulnerable to external insults [15]. Moreover, critically ill patients undergoing vasopressor and inotrope therapy for the treatment of sepsis/septic shock are at an increased risk of developing nephrotoxicity as the kidneys are more prone to ischemic damage, particularly when additional nephrotoxins such as colistin are used [16]. Chronic kidney disease also compromises renal function and often leads to the development of AKI (estimated GFR < 60 mL/min/1.73 m^2^), particularly when known nephrotoxins such as colistin are used [17].

Although the bivariate analysis showed a significant association between increased risk of nephrotoxicity and several other variables (e.g., the presence of chronic comorbid conditions such as cardiovascular disease and diabetes mellitus, hypoalbuminemia, loop diuretic treatment, and concomitant use of at least one or two nephrotoxic drugs), these associations were seen to disappear upon multivariate analysis. Previous studies have shown that chronic comorbidities and hypoalbuminemia are non-modifiable risk factors for colistin-induced AKI, and the presence of multiple chronic conditions can increase the risk of drug-induced kidney injury [2]. Recent recommendations suggest that potentially modifiable risk factors such as simultaneous exposure to other nephrotoxic drugs and the use of one or more non-specified nephrotoxins should be avoided where possible [2,14]. Wagenlehner et al., showed that the odds of developing nephrotoxicity were significantly higher among patients receiving diuretics and glycopeptides compared to those not receiving these drugs [12]. Furthermore, the rate of nephrotoxicity was seen to increase with the use of multiple nephrotoxic drugs [12].

In the current study, the bivariate analysis also showed that simultaneous use of other nephrotoxins such as amikacin, vancomycin, non-steroidal anti-inflammatory drugs, and angiotensin-converting enzyme inhibitors/angiotensin receptor blockers did not significantly favor the development of nephrotoxicity. Although these findings were in agreement with previous studies, they should be interpreted with caution due to the small number of patients examined [11,16].

### 3.2. Risk Factors for Severe AKI

In the current study, 37.7% of patients were diagnosed with stage 1 AKI while 62.3% were diagnosed with stage 2 or 3. A previous Turkish multi-center retrospective study conducted by Gunay et al., that included 149 critically ill patients receiving intravenous colistin treatment for highly resistant Gram-negative-related bacterial infections reported a high prevalence of AKI by KDIGO criteria (64.4%) [18]. Furthermore, the distribution of AKI stages was similar to the observations from the current study, with 26% and 74% of the study sample exhibiting mild (stage 1) and severe (stage 2 or 3) AKI, respectively [18]. However, Wagenlehner et al., reported a lower pooled prevalence rate of 13.1% (95% CI: 11.4–15.1) for severe nephrotoxicity in patients treated with polymyxins, and this difference could potentially be attributed to differences in the study populations examined and the criteria used to diagnose the severity of kidney injury (e.g., Risk, Injury, Failure, Loss, End-stage kidney disease (RIFLE); Acute Kidney Injury Network (AKIN); or KDIGO) [12].

The independent risk factors for AKI stages 2 and 3, in this study, included baseline hypoalbuminemia, the concomitant use of vasopressors during CMS treatment, and CMS DOT ≥ 7 days. Hypoalbuminemia has been shown to be an independent predictor of colistin nephrotoxicity irrespective of the severity [2,11,12,13]. In the current study, the bivariate analysis showed that baseline hypoalbuminemia was significantly associated with increased nephrotoxicity; however, this association was seen to disappear in the multivariate analysis. However, patients with hypoalbuminemia exhibited an increased risk of developing severe AKI (vs. stage 1 AKI). The mechanism underlying the association between hypoalbuminemia and CMS-induced kidney injury is complex and multifactorial, with the different physiological functions of albumin likely playing a significant role [19].

Subgroup analysis by time of occurrence showed that 43.5% of patients developed AKI within >7 days of CMS treatment. Furthermore, the risk of developing severe AKI increased with CMS DOT, with 39.1% of patients developing it within two days, 59.4% developing it between 3 and <7 days, and 81.7% developing it after ≥7 days of treatment (*p*-value < 0.0001). The multivariate analysis showed that CMS DOT ≥ 7 days was an independent predictor of severe AKI, and this was in agreement with the findings of previous studies that found that longer durations of treatment (and the consequent total drug exposure) were independent predictors of nephrotoxicity and functioned as a continuum instead of a single point in time [20,21,22,23,24]. Previous studies have reported a similar phenomenon of delayed nephrotoxicity developing after aminoglycoside use, wherein prolonged exposure (at least 5 to 7 days) increased the risk of toxic concentrations of aminoglycosides in the kidneys [25,26]. Wagenlehner et al., and Paquette et al., conducted a large retrospective study in Canada and found that the risk of developing AKI increased after 11 days of aminoglycoside treatment (IQR: 8–15 days) [12,26].

Although colistin dosage also plays an important role in nephrotoxicity [2], the specific effects of a loading dose on the risk of severe nephrotoxicity remain unclear. In the current study, the bivariate analysis showed that the administration of a loading dose (9 MIU) significantly increased the risk of developing stage 2 or 3 AKI; however, this association disappeared in the multivariate analysis. These findings were in agreement with the pairwise meta-analysis of studies carried out by Wagenlehner et al., wherein the odds of nephrotoxicity were found to be significantly higher in patients who received a loading dose compared to those who did not (OR = 1.833; 95% CI: 1.189–2.826; *p*-value = 0.006) [12]. Conversely, another recent meta-analysis of eight studies involving critically ill patients treated for carbapenem-resistant bacterial infections found no significant increase in the risk of nephrotoxicity (relative risk = 1.31; 95% CI: 0.90–1.91; *p*-value > 0.05) [27].

### 3.3. Prevalence of and Risk Factors for All-Cause Mortality

In the current study, all-cause mortality was recorded in more than half of the patients receiving CMS treatment, and this proportion was significantly higher among those who developed AKI (76.8%) compared to those who did not (29.4%). Furthermore, all-cause mortality was recorded in a significantly higher proportion of patients with AKI stage 2 or 3 (83.7%) compared to those with AKI stage 1 (65.4%). Independent predictors included a baseline CCI score ≥ 5, the concomitant use of vasopressors during CMS treatment, and CMS-induced AKI. These findings were in agreement with previous studies that reported an association between the development of AKI during CMS treatment and poorer prognosis, as evidenced by higher mortality rates [2]. A retrospective cohort study conducted in the United States examined 4103 patients with severe Gram-negative infections and found that 2022 (49%) patients had developed AKI. Multivariate analysis showed that the development of AKI was significantly associated with in-hospital mortality (aOR = 2.3; 95% CI: 1.9–2.7; *p*-value < 0.001) [24]. However, colistin nephrotoxicity is not the only factor contributing to renal failure and the consequent poorer outcomes since factors such as the patient’s age; the presence of comorbidities and underlying conditions; the type of systemic infection; and the causative organism that necessitated CMS treatment have all been identified as strong predictors of mortality [28,29,30,31,32]. Consequently, the establishment of a direct causal relationship between kidney injury and/or mortality and CMS treatment alone is difficult.

All these findings taken together offer additional evidence to guide clinicians when they are selecting antibiotic treatment protocols. In a real-world setting, this cohort is most likely representative of other patients necessitating last-resort antibiotic therapy like colistin for highly resistant bacteria, thus increasing the external validity of the current study. The evidence suggests that it would be prudent to avoid colistin therapy in patients > 75 years of age where possible. However, if considered unavoidable, treatment should be initiated at the early stages of infection and prior to progression to sepsis so as to minimize the need for the use of vasopressors and other nephrotoxic drugs. Furthermore, shorter treatment durations are recommended to prevent complications associated with the prolonged use of colistin (>7 days). Developing a clearer understanding of the multiple risk factors for nephrotoxicity is essential for the improvement of prognosis and treatment outcomes.

### 3.4. Importance of Applying Infection Control/Antibiotic Stewardship and Ensuring Antibiotic Equity

These findings also draw attention to two significant issues that require consideration, the first of which is the need to prevent the emergence and spread of antibiotic resistance [33]. To achieve this, a host of multilevel cost-effective interventions targeting antibiotic stewardship and the prevention and control of infections are necessary [10,34]. This could eventually lead to decreasing the use of ‘high-end’ antibiotics such as colistin, thus minimizing the risk of adverse drug effects like nephrotoxicity.

Infection prevention and control programs are mandatory in all hospitals according to the National Accreditation Standards for Hospitals in Lebanon, issued by the Lebanese Ministry of Public Health [35]. However, mandating antimicrobial stewardship programs in Lebanese hospitals is still at the beginning of the execution phase. According to these Standards, hospitals should have a process to manage and ensure proper antimicrobial prescription and limit the overuse and misuse of antimicrobials [35]. Hospitals should guide physicians and healthcare workers to restrict the overuse/misuse of antimicrobials by setting specific policies and should have indicators to measure antimicrobial consumption [35]. Despite these recommendations, many hospitals, yet not all of them, require a preauthorization for “restricted broad-spectrum antimicrobials” use by an infectious diseases physician. Nevertheless, establishing and/or implementing standalone fully functional antimicrobial stewardship programs has not yet been adopted by the vast majority of hospitals in the country.

In the hospital where the current study was conducted, antimicrobial dispensing was controlled using an institutional antimicrobial restriction policy established by the hospital’s pharmacy and therapeutics committee [10]. This policy included a list of antimicrobials that are defined as “restricted agents” [10]. It stated that only infectious disease (ID) specialists, intensivists, and chest physicians were allowed to prescribe these agents [10]. The hospital pharmacists did not dispense any “restricted antimicrobial” after the first 24 h of prescription without written consent from the formerly mentioned specialists [10]. Antimicrobial utilization strategies and institutional guidelines were unavailable at that time, and prescription was individualized and left to the judgment of each specialist [10]. By the end of 2016, an antimicrobial stewardship intervention was adopted in this hospital and was based on a prospective audit and immediate feedback to prescribers [10]. The ID physician responsible for this intervention was able to modify the choice and duration of the prescribed antimicrobial therapy after discussing the patient management plan with prescribers during daily clinical rounds [10]. Nevertheless, the antimicrobial stewardship recommendations were often not followed by pulmonologists, intensivists, and other prescribers of antimicrobial agents, which especially occurred in the critically ill patient population with severe infections and a history of recurrent antibiotic use. Antimicrobial consumption was not used as a key performance indicator where only its data were collected. The major obstacle to achieving a functional stewardship program is its acknowledgment as a standalone program with a dedicated budget, as is the case for infection control programs, rather than bits and pieces performed by different people in the absence of an allocated fund. Consequently, these facts have frequently resulted in the “uncontrolled” prescription of last-resort treatment options like CMS.

Another important issue requiring consideration is the lack of access to newer, more expensive antibiotics that are considered to be safer and more effective when attempting to eradicate highly resistant bacteria [36]. Low- and middle-income countries typically have higher rates of antibiotic resistance compared to high-income countries and, paradoxically, are also the ones facing economic challenges with regard to the provision of newer antibiotics that are effective against multi-resistant bacteria [33]. In countries with limited resources, both budget constraints in the private sector and limited government expenditure on health services contribute to inequity in access, and differential pricing of newer antibiotics based on socio-economic circumstances could be a potential solution to address this [9,36]. Implementing robust antibiotic stewardship protocols can help ensure that vital medications are deployed when they can deliver the best possible value to patients and can remain effective for the longest period possible [9].

## 4. Methods

### 4.1. Study Setting, Design, and Participants

This retrospective cohort study included patients aged >18 years who were admitted to the Makassed General Hospital (MGH), Beirut, Lebanon between January 2015 and December 2018 and received CMS for a period of 48 h or more. MGH is a 186-bed university-affiliated hospital that also functions as an acute tertiary care referral center in Beirut. CMS treatment was initiated at the discretion of the treating physician and the infectious disease consultant. The study received ethical approval from the institutional review board (IRB) at MGH (approval number: 1792019), and the requirement for informed consent was waived due to the retrospective nature of the study. In keeping with the IRB committee guidelines and regulations, only patient identification numbers were included during the data collection stage, and all cohort members were assigned unique identification numbers at a later stage to protect their privacy.

### 4.2. Data Collection

The patients’ electronic medical records were accessed from the hospital databases, and a data collection sheet was used to extract the following demographic and clinical information:

- Baseline demographic characteristics (e.g., age, gender, and body weight).
- Presence of comorbidities and underlying conditions (e.g., cardiovascular disease; respiratory disease; liver disease; cerebrovascular disease; neurological disease; diabetes mellitus; and malignancy and hematopoietic cell transplantation). Chronic kidney disease was defined as a baseline glomerular filtration rate (eGFR) < 60 mL/min/1.73 m^2^ [37]. Patients on renal replacement therapy and those with a baseline eGFR < 20 mL/min/1.73 m^2^ were excluded from the analysis. The baseline CCI was calculated to assess disease severity and risk of mortality.
- CMS dosage (in MIU) and duration of treatment (in days). The colistin available in our facility at the time of the study was labeled COLISTIMETHATE SODIQUE PANPHARMA^®^, equivalent to 1,000,000 international units of CMS and equal to 80 mg of CMS or 34 mg of colistin base activity. CMS usage up to the development of nephrotoxicity was estimated in terms of cumulative MIU and DOT prior to AKI.
- Biochemical profile (e.g., hemoglobin, white blood cell, serum albumin, and serum creatinine levels (SCr)) at baseline, peak (if AKI occurred), and end of CMS treatment. Hypoalbuminemia was defined as a serum albumin level < 3.5 g/dL [38]. EGFR was calculated using SCr and The Chronic Kidney Disease–Epidemiology Collaboration (CKD-EPI) formula [39].
- Indication for CMS use, causative organism(s), location (e.g., regular ward or critical care unit) of the patient at the time of treatment initiation, and the need for mechanical ventilation and vasopressors during the course of therapy.
- Concomitant use of potentially nephrotoxic medications including antibiotics (e.g., aminoglycosides, vancomycin, and rifampin); systemic antifungals (e.g., amphotericin B formulations); systemic antivirals (e.g., acyclovir, valacyclovir, ganciclovir, and valganciclovir); non-steroidal anti-inflammatory drugs; angiotensin-converting enzyme inhibitors; angiotensin-II receptor blockers; contrast media; diuretics; calcineurin inhibitors; vasopressors; and cytotoxic chemotherapeutic agents [40]. Combination antibiotic therapy as a treatment strategy was considered the concomitant use of at least one antibiotic with activity against Gram-negative microorganisms.
- Total all-cause mortality occurring at any stage of hospitalization.

### 4.3. AKI Severity and Renal Recovery

Acute kidney injury was defined as an increase in SCr by ≥0.3 mg/dL within 48 h or an increase in SCr to ≥1.5 times the baseline value within the last 7 days, in accordance with the KDIGO criteria [41]. The stage of severity of AKI was also defined as per the KDIGO criteria, as follows [41]:

- Stage 1 AKI: increase in SCr by ≥0.3 mg/dL in 48 h or between 1.5 and 1.9 times the baseline value within 7 days;
- Stage 2 AKI: 2.0 to 2.9 times the baseline SCr value;
- Stage 3 AKI: >3.0 times the baseline SCr value; increase in SCr ≥ 4.0 mg/dL; or commencement of renal replacement therapy regardless of previous KDIGO stage.

Renal recovery was defined as an improvement in decreased kidney function to pre-AKI baseline levels in patients who survived [32,42]. The renal recovery status at the time of hospital discharge was sub-classified into complete (i.e., patient alive, not undergoing renal replacement therapy, and exhibiting SCr level < 1.5 times the baseline value) or partial (i.e., for patients recovering from AKI stage 2 to stage 3; defined as patient alive, not undergoing renal replacement therapy, and exhibiting improvement by at least one AKI stage without restoration of SCr levels to <1.5 times the reference value) [43].

CMS-induced AKI was further divided into 3 subgroups based on the time of occurrence, as follows: (i) 2 days after initiating CMS treatment; (ii) between 3 and <7 days of treatment; and (iii) ≥7 days of treatment.

### 4.4. Statistical Analysis

All data management and analysis were carried out using the Statistical Package for Social Sciences (version 23.0; SPSS, IBM Corp.; Armonk, NY, USA) and GraphPad Prism 9.0 (GraphPad Software, Inc., San Diego, CA, USA) software for Windows. The Kolmogorov–Smirnov test was performed to assess whether the continuous variables were normally or non-normally distributed. Accordingly, normally distributed variables were expressed as mean ± standard deviation (SD) and compared using Student’s *t*-test, while non-normally distributed variables were expressed as the median and IQR and compared using the Mann–Whitney U-test. The chi-square test or Fisher’s exact test was used to compare categorical variables. The data were analyzed at a confidence level of 95%. Statistical significance in the bivariate analysis was set at *p* < 0.05. Potential factors contributing to CMS-induced AKI (regardless of severity) were identified by comparing patients who developed AKI during colistin treatment to those who did not. Predictors of AKI severity were identified by comparing patients diagnosed with CMS-induced AKI stages 2 and 3 to those diagnosed with stage 1. Survival analysis was performed using the Kaplan–Meier method, and the duration between the commencement of CMS treatment and the date of death or discharge was defined as the follow-up period. The mean/median probability of death at different time points was calculated. A log-rank test was used to compare survival by presence/absence of CMS-induced AKI and also by disease severity. Statistically significant and clinically relevant variables from the bivariate analyses were included in a forward stepwise multiple logistic regression model to allow the identification of independent risk factors for (i) CMS-induced AKI; (ii) disease severity (i.e., stages 2 and 3); and (iii) all-cause mortality. The impact and significance of each variable in the multivariate model was assessed using aOR and 95% CI. Statistical significance in the multivariable analysis was set at *p* < 0.05. Given the 34.7% risk of nephrotoxicity associated with the use of colistin (assessed using the KDIGO criteria) [12], a minimum of 203 patients were required in order to identify a difference between groups with a 95% confidence interval and a 5% margin of error. Our sample size (*n* = 298) provided the same power with a 95% confidence interval and a 5% margin of error.

## 5. Limitations and Strengths

The current study had several limitations. First, it used a retrospective study design in a single center. Second, all data were extracted from hospital records, making the verification of factors such as the suitability of indicating CMS treatment difficult. Another limitation concerned the correlation of the compromised patient outcome to AKI and its stages. As previously mentioned, this population’s baseline comorbidities, CCI scores, and critical clinical status due to severe infections caused by highly-resistant bacteria played an important role in predicting poor prognosis. Another issue of importance not explored in this paper were the potential drug–drug interactions that could have influenced AKI rates. Nevertheless, based on our findings, the concomitant use of more than one nephrotoxin did not independently affect the development of nephrotoxicity per se. Despite these limitations and to the best of our knowledge, this is the first study to examine influential factors, severity, and prognosis of CMS-induced AKI in hospital settings in Lebanon. Our sample size was enough to detect a difference between groups regarding the different study endpoints with a 95% confidence interval and a 5% margin of error. Given that colistin recipients are frequently patients with severe illnesses and infections, the cohort in this study could be expected to be representative of other likely candidates for colistin therapy in a real-world setting. The findings shed light on the interplay between geo-economics, health, and longevity in settings with limited resources.

## 6. Conclusions

In conclusion, the findings of the current study suggest that CMS treatment can lead to the development of AKI, and independent risk factors include advanced age, the presence of chronic kidney disease, and the use of vasopressors. Furthermore, the risk of developing severe AKI significantly increases with CMS DOT. These findings highlight the importance of understanding the risk factors of nephrotoxicity to improve patient prognosis and suggest that colistin therapy should be avoided in patients >75 years of age and those with pre-existing kidney diseases or hypoalbuminemia. However, when considered unavoidable, shorter treatment durations should be used along with close monitoring of renal function. Finally, these findings draw attention to the need for clinically and economically effective strategies, such as robust infection control practices and antibiotic stewardship, that can reduce the use of antibiotics such as colistin and ensure equitable access to new and relatively safe antibiotics to reduce the burden of antimicrobial resistance.

## Figures and Tables

**Table 1:** Clinical characteristics of adult inpatients who received colistimethate sodium (CMS) for ≥48 h during the study period from January 2015 to December 2018 (N = 298 patients).

Patient Characteristics	Total (N = 298 Patients) (%)
Age (years) (median, IQR)	69 (51–80)
<55	87 (29.2%)
55 to <65	45 (15.1%)
65 to <75	45 (15.1%)
≥75	121 (40.6%)
Male	131 (44.0%)
Comorbidities	
Cardiovascular Disease	122 (40.9%)
Diabetes Mellitus	94 (31.5%)
Respiratory Disease	51 (17.1%)
Chronic Kidney Disease	89 (29.9%)
Liver Disease	5 (1.7%)
Malignancy	95 (31.9%)
Hematologic Malignancy	53 (17.8%)
Solid Tumor	42 (14.1%)
Cerebrovascular Disease	102 (34.2%)
Charlson comorbidity index (CCI) upon admission	5 (2–7)
0 to 2	83 (27.9%)
3 to 4	43 (14.4%)
≥5	172 (57.7%)
Baseline serum albumin before CMS initiation (g/dL) (median, IQR)	3.01 (2.50–3.70)
<3.5	198 (66.4%)

Abbreviations: CMS = colistimethate sodium, IQR = interquartile range.

**Table 2:** Clinical indications, outcome, and other parameters related to colistimethate sodium (CMS) therapy during the study period.

Treatment Parameters	Total (N = 298 Patients) (%)
Indication of CMS	
Pneumonia	118 (39.6%)
Blood-stream infection	33 (11.1%)
Skin and soft tissue infection	17 (5.7%)
Sepsis	99 (33.2%)
Febrile Neutropenia	33 (11.1%)
Colonization	23 (7.7%)
Urinary Tract Infection	1 (0.3%)
Fever of unknown origin	11 (3.7%)
Any documented bacteria for which CMS was initiated	183 (61.4%)
Extensive drug-resistant/Carbapenem-resistant Gram-negative Bacteria	164 (55.0%)
Extensive drug-resistant/Carbapenem-resistant *Acinetobacter baumannii*	132 (44.3%)
Extensive drug-resistant/Carbapenem-resistant *Pseudomonas aeruginosa*	28 (9.4%)
*Stenotrophomonas maltophilia*	16 (5.4%)
Carbapenem-resistant Enterobacterales	15 (5.0%)
No reported organism	115 (38.6%)
Strategy of CMS use	
Monotherapy	42 (14.1%)
Combination therapy	256 (85.9%)
Patient placement when CMS was initiated	
Regular floor	172 (57.7%)
Critical care	126 (42.3%)
Development of hypotension during CMS therapy	139 (46.6%)
Need for mechanical ventilation during CMS therapy	170 (57.0%)
Cumulative CMS dose (median, IQR) (million international units)	45 (26–90)
CMS loading dose given	231 (77.5%)
CMS loading dose of 9 million international units given	199 (66.8%)
Concomitant nephrotoxic drugs	
Antimicrobials	
Amikacin	17 (5.7%)
Vancomycin	36 (12.1%)
Rifampin	2 (0.7%)
Valaciclovir	34 (11.4%)
Aciclovir	12 (4.0%)
Ganciclovir	8 (2.7%)
Valganciclovir	5 (1.7%)
Amphotericin B	14 (4.7%)
Other drugs	
Non-steroidal anti-inflammatory drugs	22 (7.4%)
Angiotensin-converting enzyme inhibitors/Angiotensin receptor blockers	19 (6.4%)
Diuretics	112 (37.6%)
Vasopressors	146 (49.0%)
Calcineurin Inhibitors (ciclosporin, tacrolimus)	5 (1.7%)
Allopurinol	7 (2.3%)
Cytotoxic chemotherapy *	11 (3.7%)
Radio-contrast agents	35 (11.7%)
Number of concomitant nephrotoxic drugs (median, IQR)	1 (1–2)
None	56 (18.8%)
≥1	242 (81.2%)
≥2	145 (48.7%)
≥3	64 (21.5%)

Abbreviations: CMS = colistimethate sodium, IQR = interquartile range N.B: *Agents included busulfan, cyclophosphamide, cytarabine, etoposide, fludarabine, and thiotepa.

**Table 3:** Independent risk factors associated with CMS-induced AKI among adult inpatients who received colistimethate sodium for ≥48 h as determined by multivariate logistic regression analysis.

Independent Risk Factor	CMS-Induced AKI	
	aOR (95% CI)	*p*-Value
Age ≥ 75 years	1.854 (1.060–3.241)	0.030
Baseline chronic kidney disease	4.849 (2.618–9.264)	<0.0001
Concomitant use of vasopressors during CMS therapy	4.305 (2.517–7.456)	<0.0001

Abbreviations: AKI = acute kidney injury, CMS = colistimethate sodium, aOR = adjusted odds ratio, CI = confidence interval.

**Table 4:** Independent risk factors associated with severe AKI among adult inpatients who received colistimethate sodium for ≥48 h as determined by multivariate logistic regression analysis.

Independent Risk Factor	Stage 2 or 3 CMS-Induced AKI	
	aOR (95% CI)	*p*-Value
Baseline hypoalbuminemia before CMS initiation (<3.5 g/dL)	2.542 (1.000–6.564)	0.049
Concomitant use of vasopressors during CMS therapy	5.472 (2.280–13.132)	<0.0001
Duration of CMS therapy prior to AKI ≥ 7 days	4.488 (1.789–11.262)	0.001

Abbreviations: AKI = acute kidney injury, CMS = colistimethate sodium, aOR = adjusted odds ratio, CI = confidence interval.

**Table 5:** Association between AKI severity and renal recovery by CMS DOT (N = 138 patients).

	Total CMS-Induced AKI (N = 138 Cases) (%)	AKI Occurring 2 Days after CMS Therapy Initiation (%)	AKI Occurring between 3 Days and <7 Days after CMS Therapy Initiation (%)	AKI Occurring at ≥7 Days of CMS Therapy Initiation (%)	*p*-Value
Total AKI	138	46 (33.3%)	32 (23.2%)	60 (43.5%)	
Severity of CMS-induced AKI					
Stage 1	52/138 (37.7%)	28/46 (60.9%)	13/32 (40.6%)	11/60 (18.3%)	<0.0001
Stage 2 or 3	86/138 (62.3%)	18/46 (39.1%)	19/32 (59.4%)	49/60 (81.7%)	

Abbreviations: AKI = acute kidney injury, CMS = colistimethate sodium.

**Table 6:** Independent risk factors associated with all-cause mortality among adult inpatients who received colistimethate sodium for ≥48 h as determined by multivariate logistic regression analysis.

Independent Risk Factor	All-Cause Mortality	
	aOR (95% CI)	*p*-Value
Charlson comorbidity index (CCI) upon admission ≥5	4.514 (2.443–8.530)	<0.0001
Concomitant use of vasopressors during CMS therapy	7.76 (4.238–14.56)	<0.0001
CMS-induced AKI	4.117 (2.231–7.695)	<0.0001

Abbreviations: AKI = acute kidney injury, CMS = colistimethate sodium, aOR = adjusted odds ratio, CI = confidence interval.

## Data Availability

The data that support the findings of this study will be shared on reasonable request to the corresponding author.

## Supplementary Materials

The following supporting information can be downloaded at: https://www.mdpi.com/article/10.3390/antibiotics12071183/s1, Table S1: Bivariate analysis of clinical characteristics and patient outcomes associated with acute kidney injury among adult inpatients who received colistimethate sodium for ≥48 h from January 2015 to December 2018 (N = 298 patients); Table S2: Bivariate analysis of clinical characteristics and patient outcomes associated with stage 2 or 3 CMS-induced AKI compared to stage 1 AKI (N = 138 patients); Table S3: Bivariate analysis of clinical characteristics associated with all-cause mortality among adult inpatients who received colistimethate sodium for ≥48 h (N = 298 patients).
