# Supplementary material for: The Prevalence and Risk Factors of Acute Kidney Injury during Colistin Therapy: A Retrospective Cohort Study from Lebanon
Source: Antibiotics (Basel). 2023 Jul 13;12(7):1183. doi: 10.3390/antibiotics12071183 (PMC10376607; doi:10.3390/antibiotics12071183)
Supplement: Supplementary file 1 [file antibiotics-12-01183-s001.zip › antibiotics-2491411-supplementary.pdf]

Supplementary Materials

Table S1: Bivariate analysis of clinical characteristics and patient outcome associated with acute kidney injury among adult inpatients who received colistimethate sodium for ≥ 48 h from January 2015 to December 2018 (N = 298 patients) (N = 298 patients)

Patient Characteristics	Total (N = 298 patients) (%)	CMS-induced AKI (N = 138 patients) (%)	No CMS-induced AKI (N = 160 patients) (%)	OR (95% CI)	*P*
Age (years) (median, IQR)	69 (51–80)	76 (60–82)	61 (39–77)		<0.0001
<55	87 (29.2%)	25 (18.1%)	62 (38.8%)	0.350 (0.204–0.599)	<0.0001
55 to <65	45 (15.1%)	17 (12.3%)	28 (17.5%)	0.662 (0.345–1.270)	0.2
65 to <75	45 (15.1%)	20 (14.5%)	25 (15.6%)	0.915 (0.484–1.732)	0.8
≥75	121 (40.6%)	76 (55.1%)	45 (28.1%)	3.133 (1.937–5.067)	<0.0001
Male	131 (44.0%)	65 (47.1%)	66 (41.3%)	1.268 (0.801–2.007)	0.3
Comorbidities					
Cardiovascular Disease	122 (40.9%)	75 (54.3%)	47 (29.4%)	2.862 (1.776–4.614)	<0.0001
Diabetes Mellitus	94 (31.5%)	54 (39.1%)	40 (25.0%)	1.929 (1.176–3.163)	< 0.01
Respiratory Disease	51 (17.1%)	31 (22.5%)	20 (12.5%)	2.028 (1.095–3.755)	0.02
Chronic Kidney Disease	89 (29.9%)	70 (50.7%)	19 (11.9%)	7.639 (4.315–13.85)	<0.0001
Liver Disease	5 (1.7%)	3 (2.2%)	2 (1.3%)	1.756 (0.289–10.662)	0.7
Malignancy	95 (31.9%)	43 (31.2%)	52 (32.5%)	0.940 (0.576–1.533)	0.8
Hematologic Malignancy	53 (17.8%)	22 (15.9%)	31 (19.4%)	0.789 (0.433–1.440)	0.4
Solid Tumor	42 (14.1%)	21 (15.2%)	21 (13.1%)	1.188 (0.618–2.282)	0.6
Cerebrovascular Disease	102 (34.2%)	47 (34.1%)	55 (34.4%)	0.986 (0.610–1.593)	1.0
Charlson comorbidity index (CCI) upon admission	5 (2–7)	6 (4–8)	4 (2–7)		<0.0001
0 to 2	83 (27.9%)	20 (14.5%)	63 (39.4%)	0.261 (0.148–0.462)	<0.0001
3 to 4	43 (14.4%)	18 (13.0%)	25 (15.6%)	0.810 (0.421–1.558)	0.5
≥5	172 (57.7%)	100 (72.5%)	72 (45.0%)	3.216 (1.978–5.231)	<0.0001

Baseline serum albumin before CMS initiation (g/dL) (median, IQR)	3.01 (2.50–3.70)	3.01 (2.38–3.50)	3.21 (2.58–3.86)		0.04
< 3.5	198 (66.4%)	102 (73.9%)	96 (60.0%)	1.889 (1.152–3.097)	0.01
Patient placement when CMS was initiated					
Regular floor	172 (57.7%)	61 (44.2%)	111 (69.4%)	0.350 (0.217–0.562)	<0.0001
Critical Care	126 (42.3%)	77 (55.8%)	49 (30.6%)	2.859 (1.778–4.599)	<0.0001
During CMS therapy					
Hypotension	139 (46.6%)	93 (67.4%)	46 (28.8%)	5.122 (3.126–8.392)	<0.0001
Mechanical ventilation	170 (57.0%)	104 (75.4%)	66 (41.3%)	4.357 (2.645–7.175)	<0.0001
Loading dose given	231 (77.5%)	109 (79.0%)	122 (76.3%)	1.171 (0.677–2.025)	0.6
Loading dose of 9 million international units given	199 (66.8%)	94 (68.1%)	105 (65.6%)	1.119 (0.689–1.816)	0.7
Concomitant Nephrotoxic Drugs					
Antimicrobials					
Amikacin	17 (5.7%)	10 (7.2%)	7 (4.4%)	1.708 (0.632–4.614)	0.3
Vancomycin	36 (12.1%)	12 (8.7%)	24 (15.0%)	0.540 (0.259–1.124)	0.1
Rifampin	2 (0.7%)	0 (0.0%)	2 (1.3%)	-	0.5
Valaciclovir	34 (11.4%)	10 (7.2%)	24 (15.0%)	0.443 (0.204–0.962)	0.04
Aciclovir	12 (4.0%)	4 (2.9%)	8 (5.0%)	0.567 (0.167–1.926)	0.4
Ganciclovir	8 (2.7%)	2 (1.4%)	6 (3.8%)	0.377 (0.075–1.901)	0.3
Valganciclovir	5 (1.7%)	0 (0.0%)	5 (3.1%)	-	0.06
Amphotericin B	14 (4.7%)	6 (4.3%)	8 (5.0%)	0.864 (0.292–2.553)	0.8
Other drugs					
Non-steroidal anti-inflammatory drugs	22 (7.4%)	11 (8.0%)	11 (6.9%)	1.173 (0.492–2.796)	0.7
Angiotensin converting enzyme inhibitors/Angiotensin receptor blockers	19 (6.4%)	10 (7.2%)	9 (5.6%)	1.311 (0.517–3.325)	0.7
Diuretics	112 (37.6%)	67 (48.6%)	45 (28.1%)	2.412 (1.493–3.897)	<0.0001
Vasopressors	146 (49.0%)	99 (71.7%)	47 (29.4%)	6.103 (3.691–10.093)	<0.0001

Calcineurin Inhibitors (ciclosporin, tacrolimus)	5 (1.7%)	2 (1.4%)	3 (1.9%)	0.770 (0.127–4.674)	1.0
Allopurinol	7 (2.3%)	2 (1.4%)	5 (3.1%)	0.456 (0.087–2.388)	0.5
Cytotoxic Chemotherapy*	11 (3.7%)	2 (1.4%)	9 (5.6%)	0.247 (0.052–1.162)	0.06
Radio-contrast agents	35 (11.7%)	11 (8.0%)	24 (15.0%)	0.491 (0.231–1.043)	0.06
Number of Concomitant Nephrotoxic Drugs (median, IQR)	1 (1–2)	2 (1–2)	1 (1–2)		<0.01
None	56 (18.8%)	18 (13.0%)	38 (23.8%)	0.482 (0.267–0.880)	0.02
≥1	242 (81.2%)	120 (87.0%)	122 (76.3%)	2.077 (1.136–3.751)	0.02
≥2	145 (48.7%)	86 (62.3%)	59 (36.9%)	2.831 (1.753–4.549)	<0.0001
≥3	64 (21.5%)	30 (21.7%)	34 (21.3%)	1.029 (0.593–1.803)	0.9
Indication of CMS					
Pneumonia	118 (39.6%)	62 (44.9%)	56 (35.0%)	1.515 (0.950–2.417)	0.08
Blood-stream infection	33 (11.1%)	17 (12.3%)	16 (10.0%)	1.264 (0.613–2.609)	0.5
Skin and soft tissue infection	17 (5.7%)	4 (2.9%)	13 (8.1%)	0.338 (0.107–1.061)	0.05
Sepsis	99 (33.2%)	48 (34.8%)	51 (31.9%)	1.140 (0.703–1.848)	0.6
Febrile Neutropenia	33 (11.1%)	10 (7.2%)	23 (14.4%)	0.465 (0.213–1.016)	0.05
Colonization	23 (7.7%)	13 (9.4%)	10 (6.3%)	1.560 (0.662–3.679)	0.3
Urinary Tract Infection	1 (0.3%)	0 (0.0%)	1 (0.6%)	-	1.0
Fever of Unknown Origin	11 (3.7%)	3 (2.2%)	8 (5.0%)	0.422 (0.110–1.624)	0.2
Documented Causative bacteria for which CMS was initiated					
Extensive drug-resistant/Carbapenem-resistant Gram-negative Bacteria	164 (55.0%)	84 (60.9%)	80 (50.0%)	1.556 (0.982–2.475)	0.06
Extensive drug-resistant/Carbapenem-resistant *Acinetobacter baumannii*	132 (44.3%)	66 (47.8%)	66 (41.3%)	1.306 (0.825–2.066)	0.3
Extensive drug-resistant/Carbapenem-resistant *Pseudomonas aeruginosa*	28 (9.4%)	17 (12.3%)	11 (6.8%)	1.903 (0.869–4.333)	0.1
Stenotrophomonas maltophilia	16 (5.4%)	6 (4.3%)	10 (6.3%)	0.682 (0.241–1.927)	0.5

Carbapenem-resistant Enterobacterales	15 (5.0%)	9 (6.5%)	6 (3.8%)	1.791 (0.621–5.164)	0.3
Strategy of CMS Use					
Monotherapy	42 (14.1%)	23 (16.7%)	19 (11.9%)	1.484 (0.770–2.859)	0.3
Combination therapy	256 (85.9%)	115 (83.3%)	141 (88.1%)	0.674 (0.350–1.298)	0.3

Abbreviations: AKI = Acute Kidney Injury, CMS = colistimethate sodium, IQR = Interquartile Range, OR = Odds Ratio, CI = Confidence Interval. N.B.
*Agents included Busulfan, Cyclophosphamide, Cytarabine, Etoposide, Fludarabine, Thiotepa

Table S2: Bivariate analysis of clinical characteristics and patient outcome associated with Stages 2 or 3 CMS-induced AKI compared to Stage 1 AKI (N = 138 patients)

	**Total CMS-Induced AKI (N = 138 patients) (%)**	**Stage 2 or 3 CMS-induced AKI (N = 86 patients) (%)**	**Stage 1 CMS-induced AKI (N = 52 patients) (%)**	**OR (95% CI)**	***P***
Age (years) (median, IQR)	76 (60–82)	77(67–83)	72 (54–81)		0.1
<55	25 (18.1%)	11 (12.8%)	14 (26.9%)	0.398 (0.165–0.961)	0.04
55 to <65	17 (12.3%)	8 (9.3%)	9 (17.3%)	0.490 (0.176–1.362)	0.2
65 to <75	20 (14.5%)	16 (18.6%)	4 (7.7%)	2.743 (0.864–8.711)	0.09
≥75	76 (55.1%)	51 (59.3%)	25 (48.1%)	1.574 (0.787–3.149)	0.2
Male	65 (47.1%)	38 (44.2%)	27 (51.9%)	0.733 (0.367–1.462)	0.4
Comorbidities					
Cardiovascular Disease	75 (54.3%)	50 (58.1%)	25 (48.1%)	1.500 (0.751–2.998)	0.3
Diabetes Mellitus	54 (39.1%)	36 (41.9%)	18 (34.6%)	1.360 (0.666–2.778)	0.4
Respiratory Disease	31 (22.5%)	20 (23.3%)	11 (21.2%)	1.129 (0.491–2.597)	0.8
Chronic Kidney Disease	70 (50.7%)	49 (57.0%)	21 (40.39%)	1.995 (0.985–3.890)	0.06
Liver Disease	3 (2.2%)	3 (3.5%)	0 (0.0%)	-	0.3
Malignancy	43 (31.2%)	23 (26.7%)	20 (38.5%)	0.584 (0.280–1.218)	0.2
Hematologic Malignancy	22 (15.9%)	12 (14.0%)	10 (19.2%)	0.681 (0.271–1.710)	0.4
Solid Tumor	21 (15.2%)	11 (12.8%)	10 (19.2%)	0.616 (0.242–1.571)	0.3
Cerebrovascular Disease	47 (34.1%)	31 (36.0%)	16 (30.8%)	1.268 (0.608–2.645)	0.6
Charlson comorbidity index (CCI) upon admission	6 (4–8)	6 (5–8)	6 (4–9)		0.1
0 to 2	20 (14.5%)	10 (11.6%)	10 (19.2%)	0.553 (0.213–1.435)	0.2
3 to 4	18 (13.0%)	11 (12.8%)	7 (13.5%)	0.943 (0.341–2.607)	0.9
≥5	100 (72.5%)	65 (75.6%)	35 (67.3%)	1.503 (0.703–3.215)	0.3
Baseline serum albumin before CMS initiation (g/dL) (median, IQR)	3.01 (2.39–3.50)	2.85 (2.30–3.43)	3.18 (2.67–3.88)		<0.01

<3.5	102 (73.9%)	69 (80.2%)	33 (63.5%)	2.337 (1.077–5.071)	0.03
Patient placement when CMS was initiated					
Regular floor	61 (44.2%)	31 (36.0%)	30 (57.7%)	0.413 (0.204–0.836)	0.01
Critical Care	77 (55.8%)	55 (64.0%)	22 (42.3%)	2.419 (1.196–4.894)	0.01
During CMS therapy					
Hypotension	93 (67.4%)	68 (79.1%)	25 (48.1%)	4.080 (1.923–8.657)	<0.0001
Mechanical ventilation	104 (75.4%)	74 (86.0%)	30 (57.7%)	4.522 (1.989–10.282)	<0.0001
Cumulative CMS dose prior to AKI (median, IQR) (million international units)	31.5 (18–61)	41.0 (21.0–72.0)	19.8 (12.3–33.9)		<0.0001
Days of CMS therapy prior to AKI (median, IQR)	4 (2–8)	6 (3–10)	2 (1–4)		<0.0001
2	46 (33.3%)	18 (20.9%)	28 (53.8%)	0.227 (0.105–0.476)	0.0001
3 to <7	32 (23.2%)	19 (22.1%)	13 (25.0%)	0.851 (0.381–1.940)	0.7
≥7	60 (43.5%)	49 (57.0%)	11 (21.2%)	4.936 (2.299–11.29)	<0.0001
Loading dose given	109 (79.0%)	73 (84.9%)	36 (69.2%)	2.496 (1.084–5.744)	0.03
Loading dose of 9 million international units given	94 (68.1%)	64 (74.4%)	30 (57.7%)	2.133 (1.025–4.440)	0.04
Concomitant Nephrotoxic Drugs					
Antimicrobials					
Amikacin	10 (7.2%)	8 (9.3%)	2 (3.8%)	2.564 (0.523–12.569)	0.3
Vancomycin	12 (8.7%)	9 (10.5%)	3 (5.8%)	1.909 (0.493–7.400)	0.5
Valaciclovir	10 (7.2%)	5 (5.8%)	5 (9.6%)	0.580 (0.160–2.109)	0.5
Aciclovir	4 (2.9%)	1 (1.2%)	3 (5.8%)	0.192 (0.019–1.898)	0.1
Ganciclovir	2 (1.4%)	1 (1.2%)	1 (1.9%)	0.600 (0.037–9.802)	1.0
Amphotericin B	6 (4.3%)	3 (3.5%)	3 (5.8%)	0.590 (0.115–3.040)	0.7
Other drugs					

Non-steroidal anti-inflammatory drugs	11 (8.0%)	6 (7.0%)	5 (9.6%)	0.705 (0.204–2.437)	0.7
Angiotensin converting enzyme inhibitors/Angiotensin receptor blockers	10 (7.2%)	7 (8.1%)	3 (5.8%)	1.447 (0.357–5.861)	0.7
Diuretics	67 (48.6%)	50 (58.1%)	17 (32.7%)	2.859 (1.391–5.878)	<0.01
Vasopressors	99 (71.7%)	74 (86.0%)	25 (48.1%)	6.660 (2.941–15.081)	<0.0001
Calcineurin Inhibitors (ciclosporin, tacrolimus)	2 (1.4%)	0 (0.0%)	2 (3.8%)	-	0.1
Allopurinol	2 (1.4%)	1 (1.2%)	1 (1.9%)	0.600 (0.037–9.802)	1.0
Cytotoxic Chemotherapy	2 (1.4%)	2 (2.3%)	0 (0.0%)	-	1.0
Radio-contrast agents	11 (8.0%)	7 (8.1%)	4 (7.7%)	1.063 (0.296–3.823)	1.0
Number of Concomitant Nephrotoxic Drugs (median, IQR)	2 (1–2)	2 (1–2)	1 (1–2)		<0.01
None	18 (13.0%)	6 (7.0%)	12 (23.1%)	1.0 (0.346–2.905)	1.0
≥1	120 (87.0%)	80 (93.0%)	40 (76.9%)	4.0 (1.373–11.470)	<0.01
≥2	86 (62.3%)	63 (73.3%)	23 (44.2%)	3.454 (1.658–7.046)	<0.01
≥3	30 (21.7%)	20 (23.3%)	10 (19.2%)	1.273 (0.568–2.994)	0.6
Renal recovery					
Reversible AKI	29 (21.0%)	12 (14.0%)	17 (32.7%)	0.334 (0.144–0.774)	<0.01
partially	15 (10.9%)	12 (14.0%)	3 (5.8%)	2.649 (0.711–9.872)	0.1
completely	14 (10.1%)	0 (0.0%)	14 (26.9%)	-	<0.0001
Irreversible AKI	109 (79.0%)	74 (86.0%)	35 (67.3%)	2.995 (1.291–6.947)	<0.01

Abbreviations: AKI = Acute Kidney Injury, CMS = colistimethate sodium, IQR = Interquartile Range, OR = Odds Ratio, CI = Confidence Interval. N.B. *Agents included Cytarabine, Etoposide, Fludarabine

Table S3: Bivariate analysis of clinical characteristics associated with all-cause mortality among adult inpatients who received colistimethate sodium for ≥ 48 hours (N = 298 patients)

Patient Characteristics	Total (N = 298 patients) (%)	All-cause Mortality (N = 153 patients) (%)	Survival (N = 145 patients) (%)	OR (95% CI)	*P*
Age (years) (median, interquartile range (IQR))	69 (51–80)	78 (66–84)	67(49–78)		<0.0001
<55	87 (29.2%)	21 (13.7%)	66 (45.5)	0.19 (0.108–0.335)	<0.0001
55 to <65	45 (15.1%)	21 (13.7%)	24 (16.6%)	0.802 (0.425–1.514)	0.5
65 to <75	45 (15.1%)	24 (15.7%)	21 (14.5%)	1.099 (0.582–2.074)	0.8
≥75	121 (40.6%)	87 (56.9%)	34 (23.4%)	4.303 (2.610–7.095)	<0.0001
Male	131 (44.0%)	62 (40.5%)	69 (47.6%)	0.750 (0.474–1.187)	0.2
Comorbidities					
Cardiovascular Disease	122 (40.9%)	85 (55.6%)	37 (25.5%)	3.649 (2.233–5.962)	<0.0001
Diabetes Mellitus	a94 (31.5%)	59 (38.6%)	35 (24.1%)	1.973 (1.196–3.254)	0,007
Respiratory Disease	51 (17.1%)	29 (19.0%)	22 (15.2%)	1.308 (0.712–2.401)	0.4
Chronic Kidney Disease	89 (29.9%)	65 (42.5%)	24 (16.6%)	3.724 (2.167–6.381)	<0.0001
Liver Disease	5 (1.7%)	5 (3.3%)	0 (0.0%)	-	0.06
Malignancy	95 (31.9%)	46 (30.1%)	49 (33.8%)	0.842 (0.517–1.372)	0.5
Hematologic Malignancy	53 (17.8%)	19 (12.4%)	34 (23.4%)	0.463 (0.250–0.856)	0.01
Solid Tumor	42 (14.1%)	27 (17.6%)	15 (10.3%0	1.857 (0.944–3.655)	0.07
Cerebrovascular Disease	102 (34.2%)	59 (38.6%)	43 (29.7%)	1.489 (0.919–2.413)	0.1
Charlson comorbidity index (CCI) upon admission	5 (2–7)	6 (5–8)	4 (2–7)		<0.0001
0 to 2	83 (27.9%)	20 (13.1%)	63 (43.4%)	0.196 (0.110–0.347)	<0.0001
3 to 4	43 (14.4%)	13 (8.5%)	30 (20.7%)	0.356 (0.177–0.714)	<0.01
≥5	172 (57.7%)	120 (78.4%)	52 (55.9%)	6.503 (3.892–10.867)	<0.0001

Baseline serum albumin before CMS initiation (g/dL) (median, IQR)	3.01 (2.50–3.70)	2.91 (2.35–3.44)	3.25 (2.66–4.05)		<0.0001
< 3.5	198 (66.4%)	113 (73.9%)	85 (58.6%)	1.994 (1.223–3.252)	<0.01
Patient placement when CMS was initiated					
Regular floor	172 (57.7%)	72 (47.1%)	100 (69.0%)	0.400 (0.249–0.642)	<0.0001
Critical Care	126 (42.3%)	81 (52.9%)	45 (31.0%)	2.500 (1.556–4.016)	<0.0001
During CMS therapy					
Hypotension	139 (46.6%)	108 (70.6%)	31 (21.4%)	8.826 (5.206–14.961)	<0.0001
Mechanical ventilation	170 (57.0%)	118 (77.1%)	52 (35.9%)	6.030 (3.630–10.015)	<0.0001
Cumulative CMS dose prior to AKI (median, IQR) (million international units)	45 (26–90)	33 (18–63.5)	27(18–36.75)		<0.01
Loading dose given	231 (77.5%)	123 (80.4%)	108 (74.5%)	1.405 (0.813–2.426)	0.2
Loading dose of 9 million international units given	199 (66.8%)	103 (67.3%)	96 (66.2%)	1.051 (0.649–1.703)	0.8
Concomitant Nephrotoxic Drugs					
Antimicrobials					
Amikacin	17 (5.7%)	7 (4.6%)	10 (6.9%)	0.647 (0.240–1.749)	0.4
Vancomycin	36 (12.1%)	16 (10.5%)	20 (13.8%)	0.730 (0.362–1.471)	0.4
Rifampin	2 (0.7%)	0 (0.0%)	2 (1.4%)	-	0.2
Valaciclovir	34 (11.4%)	9 (5.9%)	25 (17.2%)	0.300 (0.135–0.667)	<0.01
Aciclovir	12 (4.0%)	4 (2.6%)	8 (5.5%)	0.460 (0.135–1.561)	0.2
Ganciclovir	8 (2.7%)	3 (2.0%)	5 (3.4%)	0.560 (0.131–2.387)	0.5
Valganciclovir	5 (1.7%)	0 (0.0%)	5 (3.4%0	-	0.03
Amphotericin B	14 (4.7%)	5 (3.3%)	9 (6.2%)	0.511 (0.167–1.561)	0.2
Other drugs					
Non-steroidal anti-inflammatory drugs	22 (7.4%)	9 (5.9%)	13 (9.0%)	0.635 (0.263–1.533)	0.3

Angiotensin converting enzyme inhibitors/Angiotensin receptor blockers	19 (6.4%)	11 (7.2%)	8 (5.5%)	1.327 (0.518–3.396)	0.6
Diuretics	112 (37.6%)	79 (51.6%)	33 (22.8%)	3.623 (2.195–5.961)	<0.0001
Vasopressors	146 (49.0%)	117 (76.5%)	29 (20.0%)	13.000 (7.483–22.586)	<0.0001
Calcineurin Inhibitors (ciclosporin, tacrolimus)	5 (1.7%)	1 (0.7%)	4 (2.8%)	0.232 (0.026–2.100)	0.2
Allopurinol	7 (2.3%)	1 (0.7%)	6 (4.1%)	0.152 (0.018–1.282)	0.06
Cytotoxic Chemotherapy*	11 (3.7%)	2 (1.3%)	9 (6.2%)	0.200 (0.042–0.943)	0.03
Radio-contrast agents	35 (11.7%)	17 (11.1%)	18 (12.4%)	0.882 (0.436–1.786)	0.7
Number of Concomitant Nephrotoxic Drugs (median, IQR)	1 (1–2)	2 (1–2)	1 (0–2)		<0.001
None	56 (18.8%)	17 (11.1%)	39 (26.9%)	0.340 (0.183–0.628)	<0.001
≥1	242 (81.2%)	136 (88.9%)	106 (73.1%)	2.943 (1.591–5.459)	<0.001
≥2	145 (48.7%)	96 (62.7%)	49 (33.8%)	3.30 (2.030–5.362)	<0.0001
≥3	64 (21.5%)	33 (21.6%)	31 (21.4%)	1.011 (0.577–1.786)	1.0
Indication of CMS					
Pneumonia	118 (39.6%)	69 (45.1%)	49 (33.8%)	1.609 (1.007–2.572)	0.05
Blood-stream infection	33 (11.1%)	18 (11.8%)	15 (10.3%)	1.156 (0.559–2.389)	0.7
Skin and soft tissue infection	17 (5.7%)	3 (2.0%)	14 (9.7%)	0.187 (0.053–0.666)	<0.01
Sepsis	99 (33.2%)	61 (39.9%)	38 (26.2%)	1.867 (1.142–3.053)	0.01
Febrile Neutropenia	33 (11.1%)	8 (5.2%)	25 (17.2%)	0.265 (0.115–0.609)	<0.01
Colonization	23 (7.7%)	12 (7.8%)	11 (7.6%)	1.037 (0.442–2.430)	0.9
Urinary Tract Infection	1 (0.3%)	1 (0.7%)	0 (0.0%)	-	1.0
Fever of Unknown Origin	11 (3.7%)	4 (2.6%)	7 (4.8%)	0.529 (0.152–1.847)	0.3
Documented Causative bacteria for which CMS was initiated					
Extensive drug-resistant/Carbapenem-resistant Gram-negative Bacteria	164 (55.0%)	88 (57.5%)	76 (52.4%)	1.229 (0.778–1.944)	0.4

Extensive drug-resistant/Carbapenem-resistant *Acinetobacter baumannii*	132 (44.3%)	71 (46.4%)	61 (42.1%)	1.192 (0.754–1.885)	0.5
Extensive drug-resistant/Carbapenem-resistant *Pseudomonas aeruginosa*	28 (9.4%)	14 (9.2%)	14 (9.7%)	0.942 (0.430–2.068)	0.9
Stenotrophomonas maltophilia	16 (5.4%)	7 (4.6%)	9 (6.2%)	0.725 (0.263–1.999)	0.5
Carbapenem-resistant Enterobacterales	15 (5.0%)	8 (5.2%)	7 (4.8%)	1.088 (0.384–3.080)	0.9
Strategy of CMS Use					
Monotherapy	42 (14.1%)	19 (12.4%)	23 (15.9%)	0.752 (0.391–1.448)	0.4
Combination therapy	256 (85.9%)	134 (87.6%)	122 (84.1%)	1.330 (0.690–2.560)	0.4
Development of CMS-induced AKI	138 (46.3%)	106 (69.3%)	32 (22.1%)	7.964 (4.727–13.417)	<0.0001
Severity of AKI					
Stage1	52 (17.4%)	34 (22.2%)	18 (12.4%)	0.12 (0.064–0.227)	<0.0001
Stages 2 or 3	86 (28.9%)	72 (47.1%)	14 (9.7%)	8.317 (4.403–15.711)	<0.0001

Abbreviations: AKI = Acute Kidney Injury, CMS = colistimethate sodium, IQR = Interquartile Range, OR = Odds Ratio, CI = Confidence Interval. N.B. *Agents included Busulfan, Cyclophosphamide, Cytarabine, Etoposide, Fludarabine, Thiotepa
